# Preliminary Incidence and Trends of Infections with Pathogens Transmitted Commonly Through Food — Foodborne Diseases Active Surveillance Network, 10 U.S. Sites, 2016–2019

**DOI:** 10.15585/mmwr.mm6917a1

**Published:** 2020-05-01

**Authors:** Danielle M. Tack, Logan Ray, Patricia M. Griffin, Paul R. Cieslak, John Dunn, Tamara Rissman, Rachel Jervis, Sarah Lathrop, Alison Muse, Monique Duwell, Kirk Smith, Melissa Tobin-D’Angelo, Duc J. Vugia, Joanna Zablotsky Kufel, Beverly J. Wolpert, Robert Tauxe, Daniel C. Payne

**Affiliations:** ^1^Division of Foodborne, Waterborne, and Environmental Diseases, National Center for Emerging and Zoonotic Infectious Diseases, CDC; ^2^Oregon Health Authority; ^3^Tennessee Department of Health; ^4^Connecticut Emerging Infections Program; ^5^Colorado Department of Public Health and Environment; ^6^University of New Mexico, Albuquerque; ^7^New York State Department of Health; ^8^Maryland Department of Health; ^9^Minnesota Department of Health; ^10^Georgia Department of Public Health; ^11^California Department of Public Health; ^12^Food Safety and Inspection Service, U.S. Department of Agriculture, Washington, DC; ^13^Center for Food Safety and Applied Nutrition, Food and Drug Administration, Silver Spring, Maryland.

To evaluate progress toward prevention of enteric illnesses, the Foodborne Diseases Active Surveillance Network (FoodNet) of CDC’s Emerging Infections Program monitors the incidence of laboratory-diagnosed infections caused by eight pathogens transmitted commonly through food at 10 U.S. sites.* This report summarizes preliminary 2019 data and describes changes in incidence compared with that during 2016–2018. The incidence of enteric infections caused by these eight pathogens reported by FoodNet sites in 2019 continued to increase or remained unchanged, indicating progress in controlling major foodborne pathogens in the United States has stalled. *Campylobacter* and *Salmonella* caused the largest proportion of illnesses; trends in incidence varied by *Salmonella* serotype. Widespread adoption of whole genome sequencing (WGS) of bacteria has improved the ability to identify outbreaks, emerging strains, and sources of pathogens. To maximize the potential of WGS to link illnesses to particular sources, testing of isolates by clinical and public health laboratories is needed. Reductions in *Salmonella* serotype Typhimurium suggest that targeted interventions (e.g., vaccinating chickens and other food animals) might decrease human infections. Reducing contamination during food production, processing, and preparation will require more widespread implementation of known prevention measures and of new strategies that target particular pathogens and serotypes.

Members of FoodNet conduct active, population-based surveillance for laboratory-diagnosed infections caused by *Campylobacter, Cyclospora, Listeria, Salmonella,* Shiga toxin-producing *Escherichia coli* (STEC), *Shigella, Vibrio,* and *Yersinia* at 10 sites covering approximately 15% of the U.S. population (an estimated 49 million persons in 2018). FoodNet is a collaboration of CDC, 10 state health departments, the U.S. Department of Agriculture’s Food Safety and Inspection Service (USDA-FSIS), and the Food and Drug Administration (FDA). Bacterial infections are defined as isolation of the bacteria from a clinical specimen by culture or detection of pathogen antigen, nucleic acid sequences, or, for STEC,^†^ Shiga toxin or Shiga toxin genes, by a culture-independent diagnostic test (CIDT).^§^ A CIDT-positive–only bacterial infection is a positive CIDT result not confirmed by culture.^¶^ *Listeria* infections are defined as isolation of *L. monocytogenes* or detection of its nucleic acid sequences from a normally sterile site, or from placental or fetal tissue in the instance of miscarriage or stillbirth. *Cyclospora* infections are defined as detection of the parasite by microscopy using ultraviolet fluorescence or specific stains or by polymerase chain reaction. Cases with no documentation of international travel or unknown travel are considered domestically acquired infections.** The patient’s disposition at hospital discharge, or 7 days after specimen collection if not hospitalized, is attributed to the infection.

Incidence per 100,000 population was calculated by dividing the number of infections in 2019 by the U.S. Census estimates of the surveillance area population for 2018. Incidence measures include all laboratory-diagnosed infections. A negative binomial model with 95% confidence intervals (CIs) was used to estimate change in incidence during 2019 compared with that during 2016–2018, adjusting for changes in the population over time; CIs not including zero were considered statistically significant. Analyses were performed using SAS statistical software (version 9.4; SAS Institute).

Surveillance for physician-diagnosed post-diarrheal hemolytic uremic syndrome (HUS), a complication of STEC infection characterized by renal failure, thrombocytopenia, and microangiopathic anemia, is conducted by reviewing hospital discharge data and by working with a network of nephrologists and infection preventionists. This report includes HUS data for children for 2018, the most recent year for which data are available.

## Cases of Infection, Incidence, and Trends

During 2019, FoodNet identified 25,866 cases of infection, 6,164 hospitalizations, and 122 deaths (Table 1). The overall incidence per 100,000 population was highest for *Campylobacter* (19.5), followed by *Salmonella* (17.1), STEC (6.3), *Shigella* (4.8), *Cyclospora* (1.5), *Yersinia* (1.4), *Vibrio* (0.9), and *Listeria* (0.3). The respective incidences were slightly lower for domestically acquired infections (Table 2). Eighty-six percent of infections were acquired domestically, ranging from 77% for *Shigella* to 96% for *Listeria*.

**TABLE 1:** Number of laboratory-diagnosed bacterial and parasitic infections, hospitalizations, and deaths, incidence and percentage change compared with 2016–2018 average annual incidence rate, by pathogen —10 U.S. sites, Foodborne Diseases Active Surveillance Network,* 2016–2019^†^

Pathogen	2019				% Change in incidence from 2016–2018 to 2019 (95% CI)^¶^
	No. of infections	No. of hospitalizations (%)	No. of deaths (%)	Incidence^§^	
**Bacteria**					
*Campylobacter*	9,731	1,988 (20)	26 (0.3)	19.5	13 (5 to 21)
*Salmonella*	8,556	2,430 (28)	46 (0.5)	17.1	5 (−1 to 12)
STEC	3,127	660 (21)	10 (0.3)	6.3	34 (14 to 58)
*Shigella*	2,416	644 (27)	3 (0.1)	4.8	7 (−17 to 37)
*Yersinia*	681	142 (21)	4 (0.6)	1.4	153 (102 to 217)
*Vibrio*	466	131 (28)	12 (2.6)	0.9	79 (47 to 117)
*Listeria*	134	131 (98)	21 (16)	0.3	1 (−19 to 27)
**Parasite**					
*Cyclospora*	755	38 (5)	0 (0)	1.5	1,209 (708 to 2,020)
**Total**	**25,866**	**6,164 (24)**	**122 (0.5)**	**N/A**	**N/A**

**Abbreviations:** CI = confidence interval; N/A = not applicable; STEC = Shiga toxin-producing *Escherichia coli*.

* Data collected from laboratories in Connecticut, Georgia, Maryland, Minnesota, New Mexico, Oregon, Tennessee, and selected counties in California, Colorado, and New York.

^†^ Data are preliminary.

^§^ Cases per 100,000 population.

^¶^ Percentage change reported as increase or decrease. CIs not including zero are statistically significant.

**TABLE 2:** Number, percentage of all cases, and incidence of domestically acquired* laboratory-diagnosed bacterial and parasitic infections in 2019, by pathogen — 10 U.S. sites, Foodborne Diseases Active Surveillance Network,^†^ 2019^§^

Pathogen	Domestically acquired cases	
	No. (% of all cases)^¶^	Incidence**
**Bacteria**		
*Campylobacter*	8,264 (85)	16.5
*Salmonella*	7,677 (90)	15.4
STEC	2,514 (80)	5.0
*Shigella*	1,860 (77)	3.7
*Yersinia*	646 (95)	1.3
*Vibrio*	420 (90)	0.8
*Listeria*	129 (96)	0.3
**Parasite**		
*Cyclospora*	646 (86)	1.3
**Total**	**22,156 (86)**	**N/A**

**Abbreviations:** N/A = not applicable; STEC = Shiga toxin-producing *Escherichia coli*.

* Includes patients who did not have international travel in the 30 days before illness onset for *Listeria* and *Salmonella* serotypes Typhi and Paratyphi; 15 days for *Cyclospora*; and 7 days for all other pathogens and patients for whom information on international travel was not available. Information on international travel was available for 79%–89% of patients with *Campylobacter, Listeria, Salmonella, Shigella, Vibrio,* and *Yersinia* infections, and for 90% or more of patients with *Cyclospora* and STEC infection.

^†^ Data collected from laboratories in Connecticut, Georgia, Maryland, Minnesota, New Mexico, Oregon, Tennessee, and selected counties in California, Colorado, and New York.

^§^ Data are preliminary.

^¶^ Denominator is all cases, including those for which information on international travel was not available. Among patients with travel information available, the percentages of domestically acquired cases were as follows: *Campylobacter* (81%), *Cyclospora* (84%), *Listeria* (95%), *Salmonella* (87%), *Shigella* (72%), STEC (78%), *Vibrio* (89%), and *Yersinia* (94%).

** Cases per 100,000 population.

Compared with 2016–2018, the incidence in 2019 increased significantly for *Cyclospora* (1,209%), *Yersinia* (153%), *Vibrio* (79%), STEC (34%), and *Campylobacter* (13%) (Table 1). The number of bacterial infections diagnosed using a CIDT increased 32%, ranging from 18% for STEC to 253% for *Listeria*. The percentage of infections diagnosed only by CIDT, including specimens that were culture-negative and those not tested by culture, was highest for *Yersinia* (57%), followed by STEC (45%), *Campylobacter* (42%), *Vibrio* (41%), *Shigella* (40%), *Salmonella* (13%), and *Listeria* (1%). Overall, culture was attempted on 75% of positive bacterial CIDT results, ranging from 63% for *Campylobacter* to 100% for *Listeria* (Figure).

**FIGURE:**
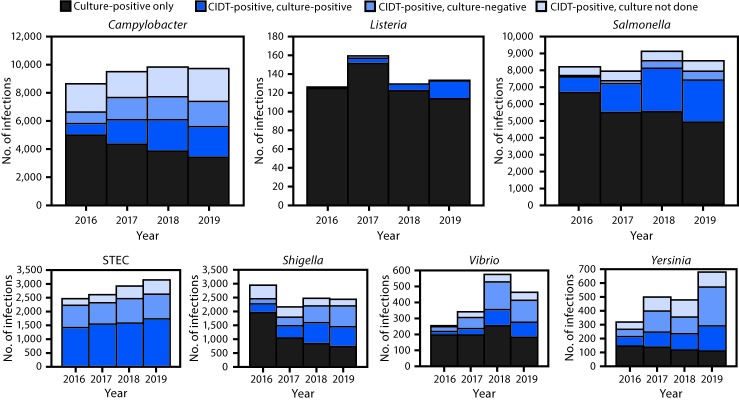
Number of infections diagnosed by culture or culture-independent diagnostic tests (CIDTs), by pathogen, year, and culture status — 10 U.S. sites, Foodborne Diseases Active Surveillance Network,* 2016–2019^†^ **Abbreviation:** STEC = Shiga toxin-producing *Escherichia coli*. * Data collected from laboratories in Connecticut, Georgia, Maryland, Minnesota, New Mexico, Oregon, Tennessee, and selected counties in California, Colorado, and New York. ^^†^^ Data for 2019 are preliminary.

Among 6,656 (90%) fully serotyped *Salmonella* isolates, the six most common serotypes were Enteritidis (2.6 per 100,000 population); Newport (1.4); Typhimurium (1.3); Javiana (1.1); I 4,[5],12:i:- (0.7); and Infantis (0.5). Compared with 2016–2018, incidence was significantly lower for Typhimurium (13% decrease; 95% CI = 1–24) and I 4,[5],12:i:- (28% decrease; 95% CI = 8–44); Infantis was significantly higher (69% increase; 95% CI = 31–118).

Among 1,725 STEC isolates, most (397; 23%) were O157, followed by O103 (305; 18%), O26 (254; 15%), and O111 (175; 10%). The incidence of STEC O157 infections (0.8 per 100,000) decreased by 20% (95% CI = 3–34), compared with that during 2016–2018; the incidence of non-O157 STEC infections (2.7) increased by 35% (95% CI = 18–56).

FoodNet identified 62 cases of post-diarrheal HUS in children (0.6 cases per 100,000) during 2018; 31 (50%) cases occurred in children aged <5 years (1.1 cases per 100,000). These rates were not significantly different from those during 2015–2017.

## Discussion

In 2019, compared with the previous 3 years, the incidence of infections caused by pathogens transmitted commonly through food increased (for *Campylobacter*, *Cyclospora*, STEC, *Vibrio*, *Yersinia*) or remained unchanged (for *Listeria, Salmonella, Shigella*). These data indicate that *Healthy People 2020* targets for reducing foodborne illness will not be met. The identification of infections that might not have been detected before adoption of CIDTs cannot explain this overall lack of progress. Better implementation of known prevention approaches and new strategies is needed to overcome the continued challenges to reducing foodborne illnesses.

Serotype Enteritidis has been the most common cause of *Salmonella* infections at FoodNet sites since 2007 and incidence has not decreased. Eggs were the major source of Enteritidis infections in the 1980s (*1*). Chicken was recognized as another important source during the late 1990s (*2*,*3*). Infantis moved from the ninth most common *Salmonella* serotype among infected persons during 1996–1998 to the sixth most common in 2019. Many infections are now caused by a new, highly resistant strain found in chicken (*4*,*5*). The incidence of some serotypes has declined. Typhimurium moved from the most common serotype during 1996–1998 to the third most common in 2019. Heidelberg, the third most common serotype during 1996–1998, is no longer among the top 20. These decreases might be partly related to the widespread practice of vaccinating chickens against Typhimurium, which shares antigens with Heidelberg (*6*). This observation, combined with a marked decline in Enteritidis infections in the United Kingdom after implementation of widespread chicken vaccination and improved farm hygiene (*7*), suggests that targeting other serotypes through poultry vaccination could be one way to reduce human illnesses in the United States.

Laboratory-diagnosed non-O157 STEC infections continue to increase. Although STEC O157 infections appear to be decreasing, outbreaks linked to leafy greens continue (*8*). Produce is also an important source for *Cyclospora*, *Listeria,* and *Salmonella* (*9*,*10*). Although adoption of syndromic panels^††^ could be contributing to the large increase in *Cyclospora*, increased exposure to this pathogen cannot be excluded. Continued implementation of FDA’s Produce Safety Rule^§§^(e.g., expanded surveillance inspections of foreign and domestically grown produce) is needed, as are innovative approaches for preventing contamination.

Advances in laboratory science continue to revolutionize enteric disease clinical diagnostics and surveillance. Many laboratories now use CIDTs to detect infections that would have previously been undiagnosed. In 2019, public health laboratories fully transitioned the standard subtyping method for clinical bacterial isolates from pulsed-field gel electrophoresis to WGS. WGS provides detailed information to more effectively recognize outbreaks, determine resistance patterns, and investigate reoccurring, emerging, and persisting strains. However, because CIDTs do not yield isolates needed to perform WGS, the full potential of these new technologies can only be realized when laboratories are fully able to culture CIDT-positive specimens.

The findings in this report are subject to at least three limitations. First, part of the observed increase in incidence is likely due to increased use of CIDTs that identify previously unrecognized infections. Changes in clinicians’ ordering practices and varying test sensitivities and specificities might also contribute to this observation. Second, changes in health care–seeking behavior, access to health services, or other population characteristics might have changed. Finally, year-to-year changes in incidence might not reflect sustained trends.

The landscape of foodborne disease continues to change, as do the methods to determine the incidence and sources of these infections. FoodNet surveillance data indicate that progress in controlling major foodborne pathogens in the United States has stalled. To better protect the public and achieve forthcoming Healthy People 2030 foodborne disease reduction goals, more widespread implementation of known prevention measures and new strategies that target particular pathogens and serotypes are needed.

**Summary**

**What is already known about this topic?**

The incidence of most infections transmitted commonly through food has not declined for many years.

**What is added by this report?**

Incidence of infections caused by *Listeria*, *Salmonella*, and *Shigella* remained unchanged, and those caused by all other pathogens reported to FoodNet increased during 2019. Infections caused by *Salmonella* serotype Enteritidis, did not decline; however, serotype Typhimurium infections continued to decline.

**What are the implications for public health practice?**

New strategies that target particular serotypes and more widespread implementation of known prevention measures are needed to reduce *Salmonella* illnesses. Reductions in *Salmonella* serotype Typhimurium suggest that targeted interventions (e.g., vaccinating chickens and other food animals) might decrease human infections. Isolates are needed to subtype bacteria so that sources of illnesses can be determined.

## Footnotes

* Connecticut, Georgia, Maryland, Minnesota, New Mexico, Oregon, Tennessee, and selected counties in California, Colorado, and New York (https://www.cdc.gov/foodnet).

† STEC infections are defined as identification of Shiga toxin or its genes by any laboratory.

§ A CIDT detects the presence of a specific antibody or antigen or the DNA of an organism.

¶ Serogroup or serotype is only available for infections confirmed by culture.

** No international travel or not known if international travel occurred within 30 days before illness onset for *Listeria, Salmonella* serotypes Typhi and Paratyphi, 15 days for *Cyclospora*, and 7 days for all other pathogens.

†† Syndromic panels are commercial CIDTs that simultaneously detect multiple pathogens associated with clinical syndromes, such as diarrheal illness.

§§ https://www.fda.gov/Food/GuidanceRegulation/FSMA/ucm334114.htm.
